# Monitoring advances including consent: learning from COVID-19 trials and other trials running in UKCRC registered clinical trials units during the pandemic

**DOI:** 10.1186/s13063-021-05225-5

**Published:** 2021-04-14

**Authors:** Sharon B. Love, Emma Armstrong, Carrie Bayliss, Melanie Boulter, Lisa Fox, Joanne Grumett, Patricia Rafferty, Barbara Temesi, Krista Wills, Andrea Corkhill

**Affiliations:** 1 MRC Clinical Trials Unit at UCL, 90 High Holborn, WC1V 6LJ London, UK; 2 Clinical Trials Research Unit, Leeds Institute of Clinical Trials Research, University of Leeds, Leeds, LS2 9JT UK; 3 Cambridge Clinical Trials Unit, Coton House Level 6, Cambridge University Hospitals NHS Foundation Trust, Addenbrooke’s Hospital, Hills Road, Cambridge, CB2 0QQ UK; 4 Nottingham Clinical Trials Unit, Building 42, University Park, Nottingham, NG7 2RD UK; 5 The Institute of Cancer Research - Clinical Trials and Statistics Unit, The Institute of Cancer Research, London, SM2 5NG UK; 6 Warwick Clinical Trials Unit, Warwick Medical School, University of Warwick, Coventry, CV4 7AL UK; 7 Northern Ireland Clinical Trials Unit, Elliot Dynes Building, Royal Hospitals, Belfast, BT12 6BA UK; 8 Manchester Clinical Trials Unit, The University of Manchester, Jean McFarlane Building, Oxford Road, Manchester, M13 9PL UK; 9 Cancer Research UK & UCL Cancer Trials Centre, UCL, 90 Tottenham Court Road, London, W1T 4TJ UK; 10 Southampton Clinical Trials Unit, University of Southampton, MP131, Southampton General Hospital, Tremona Road, Southampton, SO16 6YD UK

**Keywords:** Central monitoring, On-site monitoring, Remote monitoring, RCT, Consent, COVID-19

## Abstract

The COVID-19 pandemic has affected how clinical trials are managed, both within existing portfolios and for the rapidly developed COVID-19 trials. Sponsors or delegated organisations responsible for monitoring trials have needed to consider and implement alternative ways of working due to the national infection risk necessitating restricted movement of staff and public, reduced clinical staff resource as research staff moved to clinical areas, and amended working arrangements for sponsor and sponsor delegates as staff moved to working from home.

Organisations have often worked in isolation to fast track mitigations required for the conduct of clinical trials during the pandemic; this paper describes many of the learnings from a group of monitoring leads based in United Kingdom Clinical Research Collaboration (UKCRC) Clinical Trials Unit (CTUs) within the UK.

The UKCRC Monitoring Task and Finish Group, comprising monitoring leads from 9 CTUs, met repeatedly to identify how COVID-19 had affected clinical trial monitoring. Informed consent is included as a specific issue within this paper, as review of completed consent documentation is often required within trial monitoring plans (TMPs). Monitoring is defined as involving on-site monitoring, central monitoring or/and remote monitoring.

Monitoring, required to protect the safety of the patients and the integrity of the trial and ensure the protocol is followed, is often best done by a combination of central, remote and on-site monitoring. However, if on-site monitoring is not possible, workable solutions can be found using only central or central and remote monitoring. eConsent, consent by a third person, or via remote means is plausible. Minimising datasets to the critical data reduces workload for sites and CTU staff. Home working caused by COVID-19 has made electronic trial master files (TMFs) more inviting. Allowing sites to book and attend protocol training at a time convenient to them has been successful and worth pursuing for trials with many sites in the future.

The arrival of COVID-19 in the UK has forced consideration of and changes to how clinical trials are conducted in relation to monitoring. Some developed practices will be useful in other pandemics and others should be incorporated into regular use.

## Background

Monitoring is used within clinical trials to protect the rights and well-being of participants; to ensure data are accurate, complete and verifiable; and to confirm that the trial is being run in accordance with the protocol, with the principles of good clinical practice (GCP) and with the relevant regulatory requirements [1, 2]. The type of monitoring conducted by CTUs for a particular trial is determined by a risk assessment [3] and summarised in a trial monitoring plan.

Risk-based monitoring is often advocated [1, 4, 5]. Rather than monitoring routinely throughout the trial, the monitoring is directed at pre-defined risks to the trial, and to risks which become apparent during the active phase. The Medicines and Healthcare Products Regulatory Agency (MHRA) and others have published guidance on risk-proportionate approaches [6].

In March 2020, the COVID-19 pandemic had a global impact. Trialists in the UK had to adapt and define new ways of working. Whilst some academic sponsors had pandemic/epidemic standard operating procedures (SOPs) in place, none had been used in practice. COVID-19 trials were designed and opened at a fast rate with RECOVERY-RS trial (ISRCTN16912075) [7] recruiting the first patient just 10 days after the draft protocol. As other areas of trial conduct have had to adapt to the pandemic, so has monitoring. Two practical aspects of the pandemic are that, in order to restrict the spread of COVID-19, non-essential staff were not permitted within the hospital and research staff within hospitals were redeployed to ensure clinical care was prioritised. This led to routine on-site monitoring being impossible to undertake for most trials. Within COVID-19 trials, monitoring was still required but the use of paper-based documents (including informed consent, see Table 1) became a challenge. Hospitals mitigated against cross contamination from paper-based documents by not allowing paper to leave COVID-19 areas or delaying their release for a specific number of days. In this paper, we report the experience of the United Kingdom Clinical Research Collaboration (UKCRC) Monitoring Task and Finish Group in undertaking monitoring both within COVID-19 trials and non-COVID-19 trials being conducted during the pandemic. Members of this group were monitoring leads for nine clinical trials units (CTUs). Some of the learning is specific to COVID-19 trials, or to trials of similar infection/mode of transmission, some to non-COVID-19 trials operating during a pandemic and some ways of working could be used as routine post the COVID-19 pandemic. Informed consent is included as a specific issue within this paper, as review of the process and documentation of informed consent is often required within trial monitoring plans (TMPs). The terms on-site monitoring, centralised monitoring, remote monitoring and informed consent are defined in Table 1.

**Table 1:** Definitions

**On-site monitoring** is performed at the investigator sites at which the clinical trial is being conducted, via a physical visit by individuals from the sponsor (and/or its representatives, for example monitors and other CTU staff). It requires access to the medical records of trial participants for the purposes of source data verification/review (SDV/SDR), to confirm the accuracy of data transcription reported on the case report form (CRF), to confirm accurate reporting of all relevant clinical information (e.g. adverse events, concomitant meds), to confirm compliance with the protocol and the principles of GCP and to verify the existence of participants. On-site monitoring also usually includes site file review, verifying investigational medicinal products (receipt, storage, dispensing, accountability and destruction), review of facilities and equipment and training of site staff. On-site monitoring may be pre-planned (routine) or may occur when an issue is found at a site by central or remote monitoring (triggered).	
**Centralised monitoring** is performed in a location away from the investigator site, and often at clinical trial unit/sponsor offices. It involves an evaluation of accumulating data (or lack thereof), performed in a timely manner, supported by appropriately qualified and trained persons (e.g. data managers, statisticians, trial managers, data scientists). The aim is to mitigate specific trial risks defined in the risk assessment document which is completed before recruitment and continually reviewed during the lifetime of the trial. Data are examined by site to identify trends, outliers, anomalies, protocol deviations and inconsistencies. Concerns raised by members of the sponsor/CTU trial team discovered during their contact with the site are also taken into consideration. Centralised monitoring may be the only monitoring (or it may lead to an on-site monitoring visit). It complements and reduces the extent and/or frequency of on-site monitoring and helps distinguish between reliable data and potentially unreliable data ([1] section 5.18.3). Centralised monitoring does not require trial site staff input unless an issue is found.	
**Remote monitoring** is evaluation performed by individuals from the sponsor at a location remote from the trial site. It may include informed consent forms (ICFs) being sent to the central office to enable a number of checks to be performed with appropriate patient consent and data protection issues addressed, accountability log collection, site self-completed monitoring checklists or telephone/video monitoring calls. In some instances, remote SDV/SDR may be considered but this is dependent on sponsor procedures, site procedures and site capacity. Remote SDV/SDR may be performed by the trial site providing pseudonymised source data to the monitor, the monitor having direct access to the trial participant’s electronic medical records or using a video conferencing approach. Remote monitoring requires input from the trial site.	
**Informed consent** is a process by which a subject voluntarily confirms their willingness to participate in a particular trial, after having been informed of all aspects of the trial that are relevant to the subject’s decision to participate. Informed consent is usually documented by means of a written dual signed (patient and person taking informed consent) and dated informed consent form. Monitoring often includes checking that informed consent was correctly taken.	

## Consent

In an infectious disease setting, the patient may have the capacity to consent but there are practical limitations to capturing written evidence of this. For example, a hard copy of the patient information sheet and consent form can enter the ward, but cannot leave the ward once signed, due to the risk of infection transmission on paper documents. Various solutions around consent have been considered and incorporated into trials investigating treatments for COVID-19 as summarised in Table 2.

**Table 2:** Examples of different ways of taking consent used in COVID-19 trials

Type of consent	Comment	Pros	Cons
How consent is taken			
eConsent face-to-face	There is validated software, often linked to data management capture tools, available that can be downloaded onto a tablet for patients to provide electronic consent. Generic electronic signature products can also be used.	Informed consent can be enhanced with various features, e.g. video information, call out boxes, pictures and consent flags (dependent on the software solution). A central record can be maintained by those who require it, e.g. sponsor or CTU. Sites have access to electronic record of the informed consent form (ICF). Sites can clean electronic devices between use. Solves the problem of moving paper within infectious areas (red zones). The approach is supported by the MHRA and HRA [8]. Some evidence that eConsent improves recruitment [9].	The software has cost implications for sponsors. The availability of electronic devices in hospitals is limited or incurs a cost for sites or sponsor to purchase. Storage and charging need to be arranged. The local team may not be familiar with the software. Those monitoring will also need to be trained on this method/software.
eConsent conducted by video call	Interested participants are directed to a trial website, which outlines the study and provides the PIS. The interested participant goes through simple screening questions and those that pass are asked to provide contact details. The CTU receives the details and liaises with a clinician to call the patient. If eligible, a video call is set up with the clinician, where the PIS is discussed in full, eligibility is taken and consent is provided by the patient and clinician. On verification of consent, an email is sent to the participant which holds a link to their consent form. The clinician and CTU can access the consent form via the secure website.	Trial consent can be taken anywhere and the patient does not need to be co-located with the clinician. Site staff burden is reduced as the trial has a central group of clinicians to liaise with patients and take consent. Patients are not required to leave their homes during a pandemic so virus transmission risk reduced. Copies of the consent form are provided by email link to patients, as a record of patient consent. Evidence of consent is available to sponsor/CTU by electronic means via a secure website.	Patients may not be familiar or comfortable with sending contact details via the trial website. Patients may not be familiar with video calls. Where patients do not have access to the required technology, back up measures should be considered so that they are not excluded from the research for this reason. Additional training requirements to conventional consent. Physical examinations are not possible. High-risk trials are likely to be unsuitable, due to the virtual nature of the trial, or additional steps to confirm eligibility via patient records need to be implemented. Need a process in place to verify the participant is who they say they are—photo ID for example
Photo of written consent	Patients are provided with paper copies of the written informed consent documents on the ward. Patients/investigators sign and date consent and a photo or scan is taken of signed documents.	There are minimal cost implications, unless hospital equipment needs to be purchased. Provision of consent is in line with the usual process which local team are familiar with. Solves the problem of moving paper within infectious areas as the paper version can be processed or destroyed (and documented) in accordance with local policies.	Data protection considerations must be considered. For example: • Sites need to be compliant with their data protection policies/GDPR/SOPs, which does include photography. • Emailing identifiable images to the central office may need advance consideration of data protection issues, secure transfer and site training. Sites need access to devices to be able to take photographs. Risk that signatures are not legible/visible in photos/scans, there is a risk that the photo is lost or deleted in error resulting in a loss of evidence of consent.
Who takes the consent			
Witness [10]	A witness could be present at the time the patient gives oral consent. The witness then provides written proof of the witnessing of oral consent in a non-infected area.	There are no cost implications, a paper copy of the written informed consent document is maintained and available for monitoring purposes.	Reliant on the availability of a witness at the time of consent.
Personal legal representative (PerLR)	The use of personal legal representatives is a well-established method of consent for use in certain situations. Some COVID-19 trials have made use of this due to patients requiring mechanical ventilation. A relative or close friend is approached, either in person or via telephone, provided with the relevant information sheets and asked if they feel the participant would consent to being included in the research. If they feel the participant would be happy to take part, then a personal legal representative consent form is completed. In the context of COVID-19, these conversations should take place outside the infection area (red zone). If the participant regains capacity to consent at a later date, they are provided with the relevant information sheets and are asked to complete the consent forms indicating that they are happy for data to be used/to continue in the trial.	No cost implications. Paper copies of written informed consent are available for monitoring purposes. Preferential even when the relative or close friend was on the phone and the clinician documents their consent and the clinician signs the consent form.^a^	May be restricted to use only with patients who lack the capacity to consent due to the severity of their illness. Family may be unsure of what the participant’s wishes would be and may be reluctant to provide consent on their behalf.
Professional legal representative (ProfLR)	For patients who are critically ill and are unable to provide consent and there is either no-one suitable by virtue of their relationship to the patient or a suitable person does not wish to or is unavailable to act as the legal representative, a professional legal representative (ProfLR) may be approached. This is a medical professional caring for the patient who is unconnected to the trial. If they have no medical or ethical objections to the participant taking part, then a ProfLR consent form is completed. Again, in the context of COVID-19 research, all paperwork should be completed and stored outside the infection area (red zone). If the patient regains capacity to consent, they will be asked to complete consent to continue as detailed above.	No cost implications. Paper copies of written informed consent are available for monitoring purposes.	Restricted to use only with patients who lack the capacity to consent due to the severity of their illness.

^a^In Scotland, the ‘guardian or welfare attorney’ is the first preference, prior to the nearest relative

Legend: ‘electronic methods for seeking informed consent’ and ‘eConsent’ refer to the use of any electronic media to convey information and to see and/or document consent via an electronic device

During the development of COVID-19 trials, several CTUs utilised early and ongoing communication with the Health Research Authority (HRA) to ensure planning of the consent process was optimal and realistic. These adaptions were required due to limited resource at sites and various challenges associated with COVID-19 ‘red zones’, conscious/unconscious patients and restricted (no) face-to-face contact with relatives. There are established procedures that can be borrowed from entering adults lacking the capacity to consent [11] and minors [12] onto a clinical trial without consent.

Some COVID-19 trial designs (platform trials including more than one primary aim) were innovative in that they required consideration of several options for taking consent (Table 2).

## Monitoring without on-site visit option

In the setting of a global pandemic, access to trial sites for non-essential staff is likely to be restricted and therefore on-site visits may not be possible. In the UK COVID-19 pandemic, the HRA advised trial teams to consider what monitoring was required immediately as opposed to what could be delayed. As long as it did not add to a site’s burden, the HRA allowed alternative monitoring arrangements to be made without a protocol amendment (if site-level schedules were specified in the protocol). The risk assessment and/or monitoring plan may need to be adapted with the acceptance of some risks which would under usual circumstances be mitigated by on-site monitoring review. All file notes, monitoring mitigations and SOP deviations should be discussed with the relevant Quality Assurance (QA) team, and Monitoring Visit Reports (MVRs) must accurately state the reason why changes occurred. Updated study risk assessments should also be discussed with QA teams, chief investigators (CIs) and sponsors. Critical data points should be risk assessed for likelihood of error and whether such errors could be detected via a centralised monitoring method or simply accepted. Errors may also be picked up without the need to see the source data and can be checked by real-time review of data entered into the electronic case report form (eCRF) by CTU staff and where obvious errors are noted, or missing critical data are identified, this can be clarified directly with the trial site (routine data management). Where these risks cannot be accepted or mitigated in this way, a remote monitoring method is necessary which may include remote source data verification (SDV) or source data review (SDR). Remote monitoring can be achieved a number of ways but relies heavily on site input and is, therefore, limited by site capacity. It may be possible that sites, particularly those experienced in early phase work, may be able to perform SDV within their usual process and experience. We have experience of sites, taking on the SDV task in this way for COVID-19 trials, utilising independent staff and a robust process. Given limited resources at site currently, this option may be limited to COVID-19 trials rather than non-COVID-19 trials but offers an option in a post-COVID-19 era.

Direct access to site electronic health record (EHR) provided to the monitor away from the site enables monitoring with little site input. It can create issues around confidentiality and it is only feasible where the EHR was set up with such access in mind. Consideration needs to be given to where access takes place, for example an open plan office, public space or other locations where others who are not authorised could view sensitive information. Host organisations and sponsors (with input from their Caldicott Guardian if applicable) need to provide explicit instructions on what can be accessed where, and an agreement from the monitors that this will be complied with. Access from home can be acceptable, provided that there is somewhere private that this can be done. The device through which EHR is accessed must have adequate security, such as adequate firewalls, secure log-in and passwords etc., and must not be left unattended and accessible. The instructions should not allow printing, emailing or downloading of any records, or this should be disabled within the system [13]. Our recent experience is that this has been possible for one UK National Health Service (NHS) Trust. This method is highly effective and does not cause an increased burden for trial sites as monitors can access the data and perform review without site staff input, other than the site providing access and for addressing critical issues at the end of the review.

Pseudonymised source documents have been provided for a very small amount of data and trials. There is a high burden for sites to redact source data and this method is open to errors in redaction and copying. EMA guidance states that documents monitored in this way need to be rechecked on-site [10]. Hence, this would only be possible if there were sufficient staff time at site and only as a temporary measure.

Another remote monitoring approach is the use of video conferencing where site staff use a secure conferencing platform and screen share the health record with the monitor. Some trial sites are considering this option where it is not possible to provide the monitor with direct access to the electronic health record. As site staff will need to access the record and share their screen during the whole review, this is a high burden to sites. There is also anecdotal experience that some sites do not allow the use of video conferencing due to data protection concerns and NHS local policies; there is site variation on which systems may be permitted (if any).

Monitoring by phone using a monitoring checklist has previously been used and found useful but this method is reliant on site capacity. Source documentation is not specifically being reviewed (SDV is not possible), but the discussion can provide the opportunity to identify concerns and support the site.

Another option would be for questionnaires, site quality control checklists and essential documents to be completed by the site and sent to the monitor. This approach is limited, due to site capacity for completing checklists and scanning and emailing documents. It has previously been found useful for investigational medicinal product (IMP) accountability and acceptable to sites where pharmacy resource was not redeployed.

If one of the procedures outlined above is undertaken, then consideration also needs to be given to the process of triggering on-site monitoring visits. Where these visits will not be possible, alternative actions should be outlined such as telephone contact with site staff to assess compliance/issues and/or other types of remote monitoring activities as detailed above.

Table 3 gives more detail on phone and video conference remote monitoring.

**Table 3:** Working example of monitoring using phone and video conferencing

Action item	Aim	Monitor’s actions	Comments
1	Decrease the requirement for site-level monitoring; by implementing alternative monitoring mitigations	TC or email site staff (PI team) to ask if/when they are available for a TC or VC in place of an on-site monitoring visit and what data could be shared with the monitor ahead of this virtual meeting.	Use video conferencing if the site has the IT access. If not, then a TC. Ensure monitors have the flexibility to meet with site staff when it is convenient for the sites.
2	Prioritise patient safety, outstanding site actions and data integrity of the study primary endpoint for remote monitoring	Develop a template tracker to ensure the minimum safety and primary endpoint data are discussed in the TC/VC.	Put the questions in priority order for the TC/VC.
3	Give the site as much time as possible to prepare*	Request appropriate documents ahead of the meeting—keep this to a critical minimum.	This could include investigator site files (ISFs); delegation logs; primary endpoint pseudonymised source data.
4	Conduct a successful site TC/VC.	Review appropriate documents ahead of the meeting.	In line with preparation for all monitoring visit types
5	Prioritise the discussion with the site in case the call is cut short by technical problems or a local emergency.	Use the template tracker completed with all actions required of the site and prioritise the TC/VC discussion accordingly.	Discuss the timing of the next remote visit
6	Manage any resulting issues	Escalate any unreported SAEs, missing visits, major deviations impact on patient safety/data integrity of missing visits.	Quality, CI, PI, stats and sponsor may need to be consulted. Update study risk assessment with site-specific issues and/or availability during COVID-19 outbreak. Consider what appropriate actions, if necessary, should be undertaken
7	Complete the monitoring visit report (MVR) and site f/up letter.	As normal.	No comments.

*TC* teleconference, *VC* video conference, *CI* chief investigator, *PI* principal investigator, *SAE* serious adverse event

*Note that for COVID-19 studies, sites should be informed of and agree to the minimum data they will be asked to supply for remote monitoring, where employed

For trials in setup where remote monitoring is planned from the outset, this should be reflected in the protocol, risk assessment and informed consent.

## Monitoring without on-site or remote monitoring option

When it is not possible to undertake monitoring on-site and remote monitoring options are not possible, then focused risk-based monitoring may be considered. With this method of monitoring, the sponsor should identify the key risks involved with the trial and develop a centralised method of reviewing these risks. Primarily, the focus should be on patient safety, primary outcome measure data and any other data considered critical. An example from one CTU was to focus on data relating to patient safety. Data relating to adverse events and protocol deviations were collated and reviewed at monthly Trial Management Group (TMG) meetings where the frequency and type of events as well as the quality of the data submitted were considered. Anything which raised concern with the TMG triggered a telephone call with the site principal investigator, and further investigation was carried out if necessary. A Data Monitoring Committee (DMC) was also convened monthly with the aim of independently reviewing safety data. This method requires timely submission of data from the site so emerging triggers can be quickly identified.

Note that monitoring of all studies during the COVID-19 pandemic is likely to lead to deviations from and updates to the monitoring plan due to changing conditions.

## Minimising data

Due to the potential for placing an increased burden on participating sites that some remote monitoring methods may pose, coupled with the need to be confident in the completeness and accuracy of data, one important consideration is the quantity and complexity of the data requested.

The collection of non-essential data has long been known to increase the burden on both participating sites and sponsors, and this is now starting to be quantified. Recent data from Fougerou-Leurent et al. [14] suggest that only 13% of the data collected are critical data items and Crowley et al. [15] found 5% of items were for the primary outcome of the trial.

If the data collected can be reduced to that which is considered critical, this will reduce the burden on sites for CRF completion and sponsors for monitoring. The reduction in data collection also has wide-reaching benefits for the CTU including simplified and more rapid CRF and database development, more efficient data management and statistical analysis and, most pertinent to this review, fewer data points for which monitoring is required. RECOVERY-RS reduced the data collection to be as simple as possible, with agreement between the Trial Management Group (including the trial statistician), programming team and QA team.

If the COVID-19 pandemic can bring one benefit to clinical research in the long-term, minimising data collection would be a wide-reaching, mutually beneficial process improvement.

Although the requirement for data minimisation is well established and embedded within the Data Protection Act, 2018, the pandemic has encouraged trial development teams to consider data minimisation far more than they may have done previously. This was largely due to the clinical impact of the pandemic, when site staff were under extreme pressure, so minimising data as much as possible was essential.

## Altered data collection and retention

Limiting paper contamination within COVID-19 areas, as well as clinical trial burden due to the reduced staff resource at participating sites, has required other trial management adaptations. Examples from COVID-19 trials have included the completion of validated patient-reported outcome measures (PROMs) tools via the phone rather than patients completing the forms directly. This could be done by research staff at sites, or by CTU staff if patients have given consent for this.

COVID-19 trials also presented additional consideration when discussing where and how patients were identified and confirmed as COVID-19 positive. This was partially due to the rapidly evolving national situation around screening, availability of screening and false-negative rates and also how patients were managed once eligibility was confirmed as patients not requiring clinical care were discouraged from attending hospital settings. These issues present challenges for monitoring.

At a sponsor or central level, trial master files (TMFs) are often paper based. With many CTU staff moving to a home-based working arrangement, mitigations have needed to be made. In some instances, this has involved consideration and implementation of electronic TMF.

## Site setup changes

Although some CTUs have previously carried out site initiation visits (SIVs) using video conferencing facilities instead of on-site visits [16], during the COVID-19 pandemic, this has become the only solution for site initiation. The basic structure is similar to face-to-face SIVs with a comprehensive slide set, live presentations by the clinical and trial management teams, and an opportunity for questions. The familiar structure enables a trial team to develop materials in an established way, but then adapt the delivery to suit the current environment. Running sessions for multiple sites at a time also enables sites to benefit from shared best practice learned from other participating sites attending at the same time.

In the RECOVERY-RS trial, a large site-setup team was established, and SIVs were conducted every weekday at three time slots per day. Time slots were advertised to sites with booking details. This was cost- and time-efficient for the trial team and for site staff and enabled multiple sites to receive a SIV simultaneously. It also enabled multiple site staff to attend different SIV slots, from numerous locations, fitting in with workload and availability. With clear guidance on the meeting conduct, a separate member of the team managing the video conferencing admin, and questions posted via the ‘chat’ function, meetings ran smoothly and replicated a face-to-face meeting as much as possible.

Risk-proportionate approaches should always be used to ensure oversight of adequate experience and training of site staff, and the current situation can provide an opportunity to tailor the oversight more specifically to each individual trial. It may not always be necessary to collect curriculum vitae (CVs) and GCP certificates for all (or any) site staff, if the principal investigator (PI) or R&D department can confirm they are held on site. The PI is responsible for their research team being trained and experienced in their role. Equally, a hierarchy of study-specific training requirements may be implemented to request a core set of study training to be completed and signed off by the PI only, with PI oversight to confirm site staff have appropriate training to perform their role within the trial. Websites can be a useful tool to provide and record training and also act as a document repository, alongside a website’s traditional remit of trial promotion. Training materials can be available for sites to access electronically, with the ability to ‘sign off’ training modules online removing the need for paper copies, simultaneously providing central oversight of each site’s training status (and potentially removing the need for a separate delegation log). This facilitates local site setup, enabling work to be done at individually convenient times, without the need for printing facilities or transfer of paperwork.

## Speed of trial development

Many CTUs redirected resource to COVID-19 trial development and staff have worked at pace, contributing many hours in a short time, collaborating with many individuals and organisations (pharma, laboratories, clinicians, government) to pull together and fast track trial ideas into solid trial proposals, creating protocols in just a few weeks. Throughout the process, the MHRA and HRA have been available for early discussion, prior to submission, to ensure approvals can be swift following submission. In most cases, MHRA and HRA approvals have been issued in a matter of days including national holidays. There have also been additional steps that COVID-19 trials have needed to navigate, the primary one being Urgent Public Health (UPH) priority approval which ensured sites prioritised work on COVID-19 trials with high priority questions as deemed by the government. Due to the overwhelming demand for UPH approval, their review was sometimes conducted in parallel with MHRA and HRA review in order to maintain trial setup momentum.

Due to the speed of setup of COVID-19 studies, monitors have been required to fast track trial risk assessments and the subsequent trial monitoring plans, including considerations of mitigations due to site access restrictions and minimised site resource requirements. There was little precedence for this in the UK at the time, meaning many sponsors were ‘thinking on their feet’ to provide pragmatic solutions to emerging and evolving challenges.

Early phase trials have been required to review Safety Review Committee (SRC) processes, as usual practice is complete SDV of the data being discussed which inform dose escalations. Trial-specific mitigations have been considered (e.g. site performing SDV as described previously).

Whilst there have been significant achievements with developing complex trials in an expedited manner, there are some less positive aspects of the process. Accelerated protocol development and trial setup processes have required a substantial resource allocation, which in most cases has been delivered by experienced staff working evenings and weekends for prolonged periods. Similarly, once approved, the trials have required frequent amendment to account for the changing pandemic landscape, emerging safety information and differing processes across healthcare settings. The impact of these on the risk assessment and monitoring plans makes this challenging for sponsors, CTUs and sites to manage.

## Impact on non-COVID-19 portfolio

Many sponsors and sites suspended recruitment into trials at the start of the pandemic, and sites participating in actively recruiting trials decided if patients already recruited were able to continue treatment and follow-up. A risk-based approach was implemented and protocol processes were reviewed. Examples include clinician/patient phone calls rather than hospital clinic visits, transfer of patient care to alternative healthcare providers, provision of investigational medicinal product (IMP) to patients at home and use of validated safe-boxes for the return of samples via post. These actions have required changes to central monitoring of data, for example a metric alarm threshold for deviations would need to be increased. Often, SDV of data has been stopped with monitoring defaulting to central and remote monitoring, but this is expected to recommence as conditions permit.

Lastly, as sponsors and CTUs plan for trial restart, they should consider COVID-19 impact trial viability assessments which address if/how the trial population, interventions and outcomes have been impacted to inform if trials are able to re-open or not, and whether any changes are required. Early and open engagement with the trial site staff and the R&D office is important. Trial opening and change decisions may require trial risk assessments and the data monitoring plans to be updated. Sites need to commit to the necessary monitoring, and if the site cannot, then consideration may need to be given to keeping the site on hold to recruitment; this capacity review will often form part of the site-level risk assessment, which are likely to be required prior to re-opening [17].

## Discussion

With the arrival of COVID-19 in the UK and the need for COVID-19 trials to be rapidly created and open to recruitment, monitoring aspects of trials had to develop and adapt at speed. The announcements on clinical trial conduct from the UK Government [18], HRA [19] and EMA [10] were helpful though not exhaustive. CTU staff needed to interpret the guidance and make decisions for their own CTUs. Changes were made to the informed consent process, what data were collected and the method of setting up sites. Risks were considered within the new climate to enable monitoring without site visits or undue burden to the sites. Many of these changes could continue. For all future trials, sites could access the training at a time of their convenience and this could be recorded electronically, with the PI notified so that they can delegate tasks in an informed manner. For trials with many sites, site initiation visits could be done at regular times with personnel joining when they are able. Datasets should be minimised and patients could use on-line consent (eConsent). With monitoring, it could be recommended that we reconsider our over-reliance on on-site monitoring and more research effort could be focused on optimising central monitoring so that the site burden is minimised. As electronic patient record systems evolve, if sites could ensure that their electronic patient record systems are able to offer remote access to trial staff, restricted to specific trial participants, monitoring would be enabled. If this is not possible in the short term, site organisational risk assessments could take place to allow access to be given in the interim with data protection principles upheld.

The speed and efficiency of COVID-19 trial setup from the CTUs was complemented by the matched speed of assessment from the HRA, MHRA and UPH. It should be noted however that the intense speed of setup came at the cost of CTU staff working very intense hours, and multiple protocol and document amendments which is inefficient and undesirable outside of the pandemic situation. Some efficiency improvements could continue; examples include efficient document creation using learnings from COVID-19 trials (e.g. consent options), minimising data collection, development of database library documents and better understanding of SIV options.

Our paper only reports the experience from nine UKCRC registered CTUs and only on the monitoring aspect of trial conduct. The differences for non-CTIMP and Scottish regulations have not always been explicitly stated. However, we hope it helps others setting up COVID-19 or managing non-COVID trials during the pandemic and gives some material for consideration of changes to the conduct of clinical trials more broadly.

## Data Availability

Gavin Perkins and Danny McAuley (RECOVERY-RS Co-Cis) have agreed to referencing RECOVERY-RS.

## Abbreviations

CI: Chief investigator
CRF: Case report form
CTIMP: Clinical trial of an investigational medicinal product
CTU: Clinical trial unit
CV: Curriculum vitae
DMC: Data Monitoring Committee
eConsent: Use of any electronic media to convey information and to see and/or document consent via an electronic device
eCRF: Electronic case report form
EMA: European Medicine Agency
FDA: Food and Drug Administration
GCP: Good clinical practice
HRA: Health Research Authority
ICF: Informed consent form
ICH: International Council for Harmonisation of Technical Requirements for Pharmaceuticals for Human Use
IMP: Investigational medicinal product
ISF: Investigator site file
MHRA: Medicines and Healthcare Products Regulatory Agency
MVR: Monitoring visit report
NHS: National Health Service
PI: Principal investigator
PerLR: Personal legal representative
ProfLR: Professional legal representative
PROM: Patient-reported outcome measure
QA: Quality assurance
RCT: Randomised controlled trial
Red zone: Restricted area for patients with COVID-19
SAE: Serious adverse event
SIV: Site initiation visit
SDR: Source data review
SDV: Source data verification
SRC: Safety Review Committee
TC: Teleconference
TMF: Trial master file
TMG: Trial Management Group
TMP: Trial monitoring plan
UKCRC: United Kingdom Clinical Research Collaboration
UPH: Urgent Public Health Priority
VC: Video conference
